# Retraction: Understanding Supply-Side Implementation Barriers Affecting Child Nutrition Outcomes in the Take-Home Ration Scheme: A Qualitative Study From Madhya Pradesh, India

**DOI:** 10.7759/cureus.r228

**Published:** 2026-05-08

**Authors:** Arun Kokane, Harijith K R, Junaid Mushtaq, Nikita Rawat

**Affiliations:** 1 Community and Family Medicine, All India Institute of Medical Sciences Bhopal, Bhopal, IND

This article has been retracted by the Editor-in-Chief because the authors informed the journal that the data used in the study are owned by the Indian Council of Medical Research (ICMR), and the data were released and published without the authorization or permission of the data owner. As the authors did not possess the necessary rights or ownership to use and publish the data, the article is being retracted in accordance with Committee on Publication Ethics (COPE) guidelines.

